# Steroid-sparing benefits of biologic use in hypereosinophilic syndrome and substantial disease burden across subtypes

**DOI:** 10.3389/falgy.2025.1605397

**Published:** 2025-05-23

**Authors:** Jeremiah Hwee, Lynn Huynh, Wilson da Costa, Marc E. Rothenberg, Mei Sheng Duh, Rafael Alfonso-Cristancho

**Affiliations:** ^1^ Epidemiology, GSK, Mississauga, ON, Canada; ^2^ Analysis Group, Inc., Boston, MA, United States; ^3^ Division of Allergy and Immunology, Department of Pediatrics, Cincinnati Children’s Hospital Medical Center, University of Cincinnati School of Medicine, Cincinnati, OH, United States; ^4^ Global Real-World Evidence & Health Outcomes Research, GSK, Collegeville, PA, United States

**Keywords:** hypereosinophilic syndrome, burden of disease, biologics, mepolizumab, retrospective study, corticosteroid

## Abstract

**Background:**

Limited data exist on the burden of myeloproliferative, lymphocytic and idiopathic subtypes of hypereosinophilic syndrome (M-HES, L-HES and I-HES) and the characteristics of patients with HES receiving biologic therapies. This analysis aimed to further characterize these subtypes and explore the impact of biologics in a real-world European setting.

**Methods:**

This was a *post hoc* subgroup analysis of a retrospective, non-interventional, chart review (GSK ID: 214657) across five European countries. Index date was first clinical visit during January 2015–December 2019 (after or at time of HES diagnosis). Patients with HES aged ≥6 years with ≥1-year follow-up from index were included. Demographics, disease characteristics, diagnostic assessments, comorbidities, types of treatment, clinical manifestations, clinical outcomes and HES-related healthcare resource utilization were summarized for HES overall and subtypes. Oral corticosteroid (OCS) use and clinical manifestations/outcomes were assessed 12-months pre- and post-biologics.

**Results:**

The analysis included 280 patients with I-HES (*n* = 155), M-HES (*n* = 66), L-HES (*n* = 42) and chronic eosinophilic leukemia (*n* = 2). The most common clinical manifestations were fatigue (54.2% I-HES, 52.4% L-HES, 42.4% M-HES), skin itch (36.4% M-HES, 35.7% L-HES, 33.5% I-HES) and pain (31.0% L-HES, 30.3% M-HES, 27.1% I-HES). Biologic use was highest with L-HES (64.3%), followed by I-HES (43.9%) and M-HES (34.8%). Clinical response rates were highest for the I-HES subtype (75.5%; 66.7% L-HES, 63.6% M-HES). Hospitalizations were highest for L-HES (45.2%; 30.3% M-HES, 25.8% I-HES). The annualized rate of OCS prescriptions reduced by 56.8% (0.44–0.19 per person-year) and the proportion of patients with ≥1 clinical response increased 3.6-fold (6.5%–23.4%) between the pre- and post-biologics periods.

**Conclusions:**

All HES subtypes had a substantial disease burden and were commonly associated with fatigue, skin itch and pain. I-HES appeared to be more responsive to treatment than L-HES and M-HES. Biologic use for HES led to more patients experiencing clinical responses and was OCS-sparing.

## 1 Introduction

Hypereosinophilic syndromes (HES) are rare and heterogenous blood disorders characterized by persistent hypereosinophilia (>1,500 cells/µl) and eosinophilic tissue infiltration leading to organ damage (1–3) HES prevalence has been estimated to be 1.5 cases per 100,000 people in Europe (4), but regional estimates vary (North America: 0.32–6.3 cases per 100,000; UK: 0.15–0.89 cases per 100,000) (5, 6). HES is diagnosed when known causes of secondary hypereosinophilia are excluded (1, 3, 7, 8) and can be classified into several subtypes, including myeloproliferative, lymphocytic and idiopathic variants (M-HES, L-HES and I-HES, respectively), based on distinct molecular characteristics (1, 3, 7, 9).

M-HES is diagnosed in patients whose hypereosinophilia is caused by a defined myeloid malignancy or who have clinical characteristics consistent with one (7). L-HES is a form of reactive HES, diagnosed when patients present with an aberrant immunophenotype in ≥1 *T*-cell subset, increased type 2 cytokine production and signs of HES-related organ damage; clonal T-cell receptor gene rearrangement may or may not be evident (3). I-HES is a diagnosis of exclusion, made when the criteria for HES are fulfilled and all known primary and secondary causes of hypereosinophilia have been excluded (1).

HES-related disease flares, defined as a period of worsening of HES-related symptoms, have a significant adverse impact on health and quality of life and may be life-threatening. The goals of HES treatment are to control disease symptoms and minimize tissue damage (10). Therapy typically relies on high-dose maintenance oral corticosteroids (OCS), while immunosuppressant and/or cytotoxic therapies may be added for OCS-resistant HES (11). OCS can be associated with adverse effects and may be less effective in patients with M-HES and L-HES (11–13). In the first-line setting, M-HES may also be treated with tyrosine kinase inhibitors such as imatinib (14). Several biologics have demonstrated reductions in circulating eosinophils resulting in clinical benefits (14, 15). Mepolizumab is a biologic targeting interleukin (IL)-5, which is approved in Europe and the US for inadequately-controlled HES without an identifiable non-hematologic cause (16, 17). In a Phase III trial, mepolizumab was associated with a statistically significant reduction in the proportion of patients experiencing flares, or having to withdraw from the trial, vs. placebo (18). Targeted therapies, such as mepolizumab, can contribute to OCS-sparing strategies and could improve clinical outcomes for patients (19–21).

A retrospective, non-interventional study of patients with HES from France, Germany, Italy, Spain and the UK, identified a substantial disease burden including comorbidities such as asthma, anxiety or depression, hypertension and chronic sinusitis with nasal polyps (22). Additionally, high rates of healthcare resource utilization (HCRU) were reported, with approximately 30% of patients requiring hospitalization, 26% requiring emergency department visits and 87% requiring outpatient visits for HES-related reasons over a median follow-up of 2.4 years (22). These findings highlight the need for optimized HES management strategies.

Given that HES is a rare disorder there are limited data available on clinical outcomes and treatment patterns for patients and there are very few published studies reporting such data in patients with M-HES, L-HES or I-HES subtypes (9, 13, 23, 24). There is some evidence that symptoms differ according to subtype. For example, I-HES appears to be particularly associated with left-ventricular abnormalities, respiratory issues and gastrointestinal symptoms; M-HES with splenomegaly, anemia and fatigue; and L-HES with skin lesions/rashes and gastrointestinal symptoms (9). Additionally, there are limited data globally from clinical trials or real-world usage of emerging biologic therapies for HES and their clinical benefits, including OCS-sparing (15, 18, 19, 25, 26). Therefore, this *post hoc* subgroup analysis aimed to characterize the burden of HES by disease subtype, to gain better understanding of the characteristics of patients with HES receiving biologic therapies and to explore the clinical impact of biologic therapy in a real-world European setting.

## 2 Materials and methods

### 2.1 Study design and population

These were *post hoc* subgroup analyses of a retrospective, non-interventional, longitudinal, physician panel-based chart review study (GSK ID: 214657) of patients diagnosed with HES across five European countries (Germany, Italy, France, Spain and the UK), the methodology of which has been reported previously (22). Briefly, diagnosis date was defined as the date of HES diagnosis and index date was defined as the first clinical visit for any reason occurring between January 2015 and December 2019 (patients could have an existing diagnosis of HES or be newly diagnosed at the index date). For patients diagnosed before the index date, a pre-index period was utilized from which patient demographics and baseline clinical characteristics were identified. Follow-up included the period from index date to the earliest occurrence of death, loss to follow-up or end date of chart abstraction. The last date of follow-up was 14 July, 2021. The study included patients with a physician-confirmed HES diagnosis aged ≥6 years at time of diagnosis with ≥1-year follow-up from index, except where follow-up ended due to death.

### 2.2 Data source and data collection

Longitudinal patient-level data were obtained from demographically and geographically diverse, nationally representative populations. Physicians (*N* = 121) were the primary healthcare provider for ≥1 eligible patient with HES, with access to their medical records. Physicians abstracted medical charts of randomly selected patients. Physicians were from different practice settings (academic or community-based) and included targeted specialties (allergy, hematology, immunology, internal medicine, pulmonology and rheumatology) from each participating country. Only anonymized data were collected. Physicians were blind to the identity of the study sponsor and vice versa.

Demographic and baseline characteristic information were obtained from patient charts from the pre-index period (between HES diagnosis and the index date for patients diagnosed before index; these characteristics were also collected for patients diagnosed at index). Clinical manifestations and outcomes of HES and HCRU were assessed from the index date until the end of follow-up. Comorbid conditions and treatment patterns were assessed from HES diagnosis until end of follow-up, unless otherwise stated.

### 2.3 Subgroup analysis of patient characteristics and outcomes by HES subtype

Data were summarized for the overall HES population and M-HES, L-HES and I-HES subtypes (defined *post hoc*) as follows: patient demographics and disease characteristics (including age at diagnosis, age at index and disease duration), diagnostic assessments, comorbidities, types of treatment (e.g., OCS, immunosuppressant/cytotoxic agents, biologics or other therapies), clinical manifestations, clinical outcomes and HES-related HCRU. Clinical outcomes included the occurrence of flares, defined as a worsening of HES-related symptoms or blood eosinophil counts requiring therapy escalation (dose increase or additional/new therapy) and responses to treatment. A complete response was defined as physician-reported improved or resolved symptoms and a normal eosinophil count (≤500 cells/ml) (27). Partial response reflected improved symptoms and eosinophil count that was improved but was not in the reference range and required more therapy.

### 2.4 Subgroup analysis of patients receiving biologic therapy

Demographics, disease characteristics, comorbidities and HCRU were summarized for the subgroup of patients who had received biologic treatment between HES diagnosis and end of follow-up. For patients with a non-missing date for ≥1 biologics prescription pre- and post-biologic initiation periods were defined. The pre-biologics period was defined as 12 months before and including the initiation of biologics. Only events and person-years after HES diagnosis were included. The post-biologics period was defined as 12 months after the initiation of biologics, or until death or end of follow-up. The following outcomes were summarized for ≤12 months pre- and post- biologics initiation: OCS use (annualized rate of prescriptions and proportion of patients receiving ≥1 prescription), clinical manifestations (proportion of patients with manifestation) and clinical outcomes (flares and clinical response to treatment).

### 2.5 Sample size and statistical analysis

The subgroup analyses were defined *post hoc*. All study outcomes were summarized using descriptive statistics and no comparative testing was performed. Real-world flare-free survival (FFS) was assessed over the 6 years following the initial diagnosis date (6-year restricted mean survival time) using Kaplan–Meier analysis. Data analysis was performed using SAS Enterprise Guide Version 7.15 (SAS Institute Inc., Cary, NC, USA).

## 3 Results

### 3.1 Patient characteristics and outcomes by HES subtype

Of the 280 patients for whom data were collected, 265 had a physician-defined subtype and 15 had unknown disease subtype. Of those with a physician-defined subtype, 155 had I-HES, 66 had M-HES, 42 had L-HES and 2 had chronic eosinophilic leukemia. Overall patient demographics and physician characteristics have been published previously (22). HES subtype distribution varied between countries, although in all countries most patients had I-HES [51.9% in Spain (lowest), 61.5% in Italy (highest); 55.4% overall]. The highest proportion of patients with M-HES was in Spain (34.6%; 23.6% overall) and with L-HES was in the UK (22.6%; 15% overall).

A higher proportion of patients with M-HES were male (75.8%) and were older at diagnosis (mean age 47.0 years) than in the overall population (65.0% and 42.4 years, respectively) and other subtypes (Table 1). The median number of diagnostic assessments was similar across HES subtypes; however, types of testing performed varied according to disease subtype (Table 1). For example, allergy testing (83.3%) and kidney function testing (95.2%) were performed most frequently among patients with L-HES, whereas bone marrow aspiration and biopsy (80.3%) and molecular genetic testing (71.2%) were more frequent in patients with M-HES. Median duration of HES (2.5–2.9 years) and length of follow-up (2.3–2.5 years) were similar across subtypes. The highest median [interquartile range (IQR)] blood eosinophil count was observed in the M-HES subtype [2,300.0 (850.0, 4,500.0) cells/µl]; counts in L-HES and I-HES were 1,570.0 and 1,890.0 cells/µl, respectively (Table 1).

**Table 1:** Baseline patient demographics and disease characteristics across HES subtypes (22).

Patient baseline characteristics	Overall^a^ (*N* = 280)	Myeloproliferative (*N* = 66)	Lymphocytic (*N* = 42)	Idiopathic (*N* = 155)
Patient country, *n* (%)				
France	61 (21.8)	16 (24.2)	11 (26.2)	33 (21.3)
Germany	53 (18.9)	10 (15.2)	8 (19.0)	30 (19.4)
Italy	52 (18.6)	9 (13.6)	4 (9.5)	32 (20.6)
Spain	52 (18.6)	18 (27.3)	5 (11.9)	27 (17.4)
UK	62 (22.1)	13 (19.7)	14 (33.3)	33 (21.3)
Age at HES diagnosis, years, mean (SD)	42.4 (16.2)	47.0 (16.0)	40.0 (17.9)	40.7 (15.6)
Age on index date^b^, years, mean (SD)	43.7 (15.8)	47.7 (15.7)	40.8 (17.9)	42.2 (15.1)
6–11 years, *n* (%)	9 (3.2)	2 (3.0)	1 (2.4)	6 (3.9)
12–17 years, *n* (%)	9 (3.2)	1 (1.5)	5 (11.9)	3 (1.9)
Male, *n* (%)	182 (65.0)	50 (75.8)	28 (66.7)	98 (63.2)
Year of diagnosis, *n* (%)				
2014 or before	32 (11.4)	6 (9.1)	4 (9.5)	20 (12.9)
2015–2019	248 (88.6)	60 (90.9)	38 (90.5)	135 (87.1)
Disease duration^c^, years, median (IQR)	2.7 (1.8, 4.1)	2.7 (1.9, 3.8)	2.5 (1.7, 4.1)	2.9 (1.8, 4.6)
Length of follow-up^d^, years, median (IQR)	2.4 (1.6, 3.6)	2.5 (1.7, 3.2)	2.3 (1.6, 3.5)	2.5 (1.6, 3.7)
Blood eosinophil count, cells/µl, median (IQR)^e^	1,900.0 (690.0, 3,500.0)	2,300.0 (850.0, 4,500.0)	1,570.0 (485.0, 5,500.0)	1,890.0 (790.0, 3,000.0)
Number of diagnostic assessments, median (IQR)^f^	10.0 (6.0, 12.0)	10.0 (6.0, 14.0)	10.0 (7.0, 12.0)	10.0 (6.0, 12.0)
Diagnostic assessments, *n* (%)^g^				
Absolute blood eosinophil count	261 (93.2)	61 (92.4)	39 (92.9)	146 (94.2)
Allergy tests to diagnose environmental or food allergies^h^	195 (69.6)	42 (63.6)	35 (83.3)	109 (70.3)
Blood tests to screen for autoimmunity	201 (71.8)	46 (69.7)	30 (71.4)	115 (74.2)
Bone marrow aspiration and biopsy	166 (59.3)	53 (80.3)	22 (52.4)	86 (55.5)
Imaging scans of affected organs^i^	201 (71.8)	50 (75.8)	33 (78.6)	110 (71.0)
Kidney function tests	237 (84.6)	55 (83.3)	40 (95.2)	130 (83.9)
Liver function tests	225 (80.4)	51 (77.3)	35 (83.3)	127 (81.9)
Molecular genetic tests to detect *FIP1L1*–*PDGFRA* or other chromosomal abnormalities^j^	147 (52.5)	47 (71.2)	18 (42.9)	78 (50.3)
Skin or other tissue biopsy	122 (43.6)	26 (39.4)	22 (52.4)	67 (43.2)
Tests to detect parasitic infection	142 (50.7)	40 (60.6)	15 (35.7)	85 (54.8)
Screening for ABPA	92 (32.9)	27 (40.9)	17 (40.5)	46 (29.7)
Other methods	3 (1.1)	2 (3.0)	1 (2.4)	0 (0.0)

ABPA, allergic bronchopulmonary aspergillosis; CT, computed tomography; EOF, end of follow-up; HES, hypereosinophilic syndrome; IQR, interquartile range; MRI, magnetic resonance imaging; SD, standard deviation.

^a^ Overall (*N* = 280) included patients with myeloproliferative (*N* = 66), lymphocytic (*N* = 42), idiopathic (*N* = 155), other (*N* = 2) and unknown (*N* = 15) disease subtypes.

^b^ Index date was defined as the date of a patient's earliest visit with their physician between January 2015 and December 2019 on or after the patient's HES diagnosis.

^c^ Disease duration was calculated as the time between HES diagnosis and EOF (i.e., last physician encounter or death).

^d^ Length of follow-up was calculated as the time between index date and EOF (i.e., last physician encounter or death).

^e^ Most recent documented lab test value between diagnosis and index date. Blood eosinophil counts values available for *n* = 241, *n* = 56, *n* = 38, *n* = 133 patients from the overall, M-HES, L-HES and I-HES subtypes respectively.

^f^ All categories of diagnostic assessment were counted for this statistic. For example, if a physician indicated that a patient had an imaging scan of affected organs, and then further specified that the patient had an abdominal CT and a chest radiograph, this would count as two diagnostic assessments.

^g^ Categories of diagnostic assessments were not mutually exclusive.

^h^ Includes blood tests and allergy skin tests.

^i^ Includes abdominal CT, chest radiograph, chest CT, sinus CT, echocardiogram, cardiac MRI, other imaging.

^j^ Examples of other chromosomal abnormalities include *BCR-ABL1, JAK2 V617F, KIT D816V*, clonal *T*-cell receptor rearrangements and karyotyping.

Asthma, anxiety or depression, nasal polyps and hypertension were the most common comorbidities across subtypes (Table 2). Asthma was most common in I-HES (52.3%) and less common in M-HES and L-HES subtypes (25.8%–47.6%, respectively). Anxiety or depression affected more patients with M-HES (48.5%) than L-HES (33.3%) or I-HES (34.2%). Nasal polyps were most common with I-HES (38.7%) and less common with the other subtypes (23.8%–27.3%); while rates of hypertension were reported in around one-third of patients (31.6%–36.4%) across subtypes (Table 2).

**Table 2:** Patient comorbidities across HES subtypes (assessed between HES diagnosis and end of follow-up).

Comorbidities,^b^ *n* (%)	Overall^a^ (*N* = 280)	Myeloproliferative (*N* = 66)	Lymphocytic (*N* = 42)	Idiopathic (*N* = 155)
Asthma	126 (45.0)	17 (25.8)	20 (47.6)	81 (52.3)
Anxiety or depression	102 (36.4)	32 (48.5)	14 (33.3)	53 (34.2)
Nasal polyps^c^	91 (32.5)	18 (27.3)	10 (23.8)	60 (38.7)
Hypertension	91 (32.5)	24 (36.4)	14 (33.3)	49 (31.6)
Vasculitis	47 (16.8)	8 (12.1)	8 (19.0)	30 (19.4)
Obesity	44 (15.7)	12 (18.2)	5 (11.9)	23 (14.8)
Lower respiratory disease(s)^d^	39 (13.9)	12 (18.2)	6 (14.3)	19 (12.3)
Diabetes	35 (12.5)	12 (18.2)	8 (19.0)	12 (7.7)
Osteoporosis	31 (11.1)	10 (15.2)	4 (9.5)	17 (11.0)
Liver disease	24 (8.6)	10 (15.2)	4 (9.5)	9 (5.8)
Glomerulonephritis	21 (7.5)	6 (9.1)	6 (14.3)	8 (5.2)
Any cancer	20 (7.1)	11 (16.7)	5 (11.9)	3 (1.9)
Cerebrovascular disease	9 (3.2)	1 (1.5)	3 (7.1)	3 (1.9)
Rheumatoid arthritis	8 (2.9)	2 (3.0)	4 (9.5)	1 (0.6)
Other^e^	8 (2.9)	2 (3.0)	2 (4.8)	3 (1.9)

COPD, chronic obstructive pulmonary disease; EOF, end of follow-up; HES, hypereosinophilic syndrome.

^a^ Overall (*N* = 280) included patients with myeloproliferative (*N* = 66), lymphocytic (*N* = 42), idiopathic (*N* = 155), other (*N* = 2) and unknown (*N* = 15) HES subtypes.

^b^ Clinical manifestations, comorbidities and cancer diagnoses were assessed between HES diagnosis and EOF (i.e., last physician encounter or death).

^c^ The nasal polyps option on the case report form did not specify whether this was chronic rhinosinusitis with nasal polyps or otherwise.

^d^ Other than asthma and COPD.

^e^ Other reported comorbidities included allergy (1), angioedema (1), colitis (1), esophagitis (1), eosinophilic gastroenteritis (1), restriction (1) and rhinitis (2).

**Figure 1:**
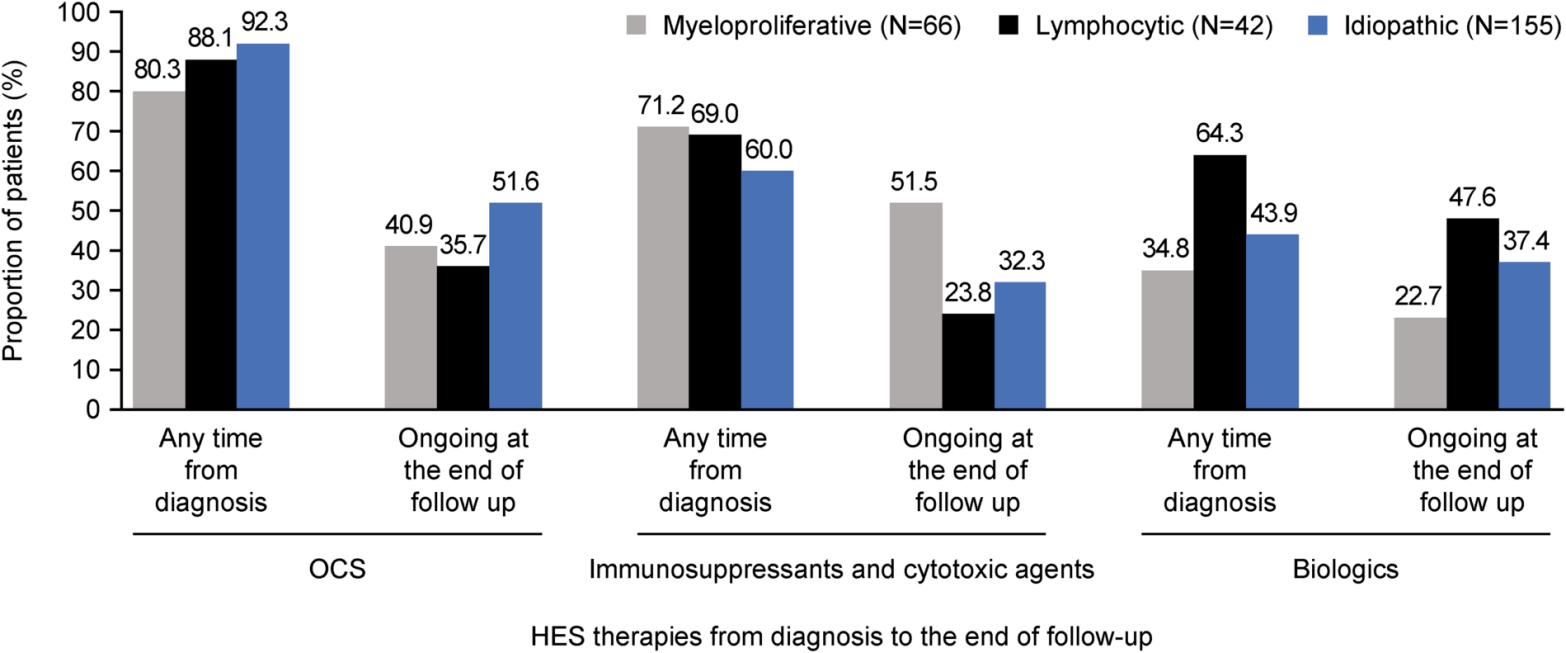
Summary of proportion of patients with OCS, immunosuppressive/cytotoxic and biologic use at any time from diagnosis and ongoing at end of follow-up, across HES subtypes. HES, hypereosinophilic syndrome; OCS, oral corticosteroids.

Mean [standard deviation (SD)] time from diagnosis to initiation of HES therapy was shortest for M-HES [0.4 (1.1) years] and longest for L-HES [1.0 (2.2) years; Table 3]. OCS were received by 80.3%–92.3% of patients across all subtypes and were most commonly used among patients with I-HES; however, the mean (SD) maximum daily OCS dose was lowest in the I-HES subtype [28.4 (19.3) mg] and highest with the M-HES subtype [38.6 (19.0) mg; Figure 1; Table 3]. The use of immunosuppressive and cytotoxic therapies was most common among the M-HES (71.2%) and L-HES (69.0%) subtypes. This included the targeted therapy imatinib, which was used by 39.4% of patients with M-HES, 2.4% with L-HES and 17.4% with I-HES (Table 3). Biologic use was highest in patients with L-HES (64.3%), followed by I-HES (43.9%) and M-HES (34.8%). The most common biologic therapies were mepolizumab (15.4%), benralizumab (12.1%) and rituximab (12.1%). Mean (SD) time from HES diagnosis to biologic initiation ranged from 2.7 (3.2) years for L-HES to 3.0 (3.6) years for I-HES (Table 3). The most common therapies reported as ongoing at end of follow-up were OCS in I-HES, immunosuppressants/cytotoxic therapies in M-HES and biologic therapies in L-HES (Figure 1; Supplementary Table 1).

**Table 3:** Detailed treatment patterns across HES subtypes.

HES therapies (assessed between diagnosis and EOF)	Overall^a^ (*N* = 280)	Myeloproliferative (*N* = 66)	Lymphocytic (*N* = 42)	Idiopathic (*N* = 155)
Number of distinct HES therapies used, mean (SD) [median]	2.5 (1.5) [2.0]	2.4 (1.7) [2.0]	3.0 (1.7) [3.0]	2.4 (1.3) [2.0]
Time from diagnosis to initiation of therapy (years), mean (SD) [median]	0.7 (1.8) [0.0]	0.4 (1.1) [0.0]	1.0 (2.2) [0.1]	0.6 (1.7) [0.0]
Time from diagnosis to initiation of biologics (years), mean (SD) [median]	3.1 (4.4) [1.8]	2.8 (3.6) [1.9]	2.7 (3.2) [1.6]	3.0 (3.6) [2.0]
HES therapies by treatment category				
Oral corticosteroids, *n* (%)	250 (89.3)	53 (80.3)	37 (88.1)	143 (92.3)
Patients with a reported maximum daily dose for maintenance therapy,^b^ *n* (%)	190 (76.0)	40 (75.5)	28 (75.7)	112 (78.3)
Maximum daily dose across all oral corticosteroids (mg), mean (SD) [median]	31.7 (19.1) [25.0]	38.6 (19.0) [40.0]	33.6 (16.9) [30.0]	28.4 (19.3) [20.0]
Duration across all oral corticosteroids (months), mean (SD) [median]	23.5 (27.8) [18.3]	22.6 (18.8) [21.1]	15.3 (14.2) [12.7]	28.0 (34.1) [19.8]
Prednisone or prednisolone, *n* (%)	200 (71.4)	42 (63.6)	27 (64.3)	118 (76.1)
Methylprednisolone, *n* (%)	46 (16.4)	8 (12.1)	9 (21.4)	23 (14.8)
Cortisone, *n* (%)	14 (5.0)	6 (9.1)	3 (7.1)	5 (3.2)
Any immunosuppressants or cytotoxic agents, *n* (%)	178 (63.6)	47 (71.2)	29 (69.0)	93 (60.0)
Imatinib mesylate	57 (20.4)	26 (39.4)	1 (2.4)	27 (17.4)
Azathioprine	40 (14.3)	3 (4.5)	6 (14.3)	29 (18.7)
Methotrexate	29 (10.4)	7 (10.6)	5 (11.9)	13 (8.4)
Hydroxyurea	26 (9.3)	4 (6.1)	6 (14.3)	15 (9.7)
Cyclophosphamide	17 (6.1)	5 (7.6)	5 (11.9)	7 (4.5)
Other^c^	60 (21.4)	18 (27.3)	15 (35.7)	27 (17.4)
Most common biologics, *n* (%)	123 (43.9)	23 (34.8)	27 (64.3)	68 (43.9)
Mepolizumab	43 (15.4)	11 (16.7)	10 (23.8)	20 (12.9)
Benralizumab	34 (12.1)	4 (6.1)	11 (26.2)	18 (11.6)
Rituximab	34 (12.1)	7 (10.6)	8 (19.0)	16 (10.3)
Alemtuzumab	22 (7.9)	7 (10.6)	4 (9.5)	11 (7.1%)
Dupilumab	22 (7.9)	4 (6.1)	3 (7.1)	15 (9.7)
Other^d^	26 (9.3)	4 (6.1)	8 (19.0)	14 (9.0)

EOF, end of follow-up; HES, hypereosinophilic syndrome; SD, standard deviation.

^a^ Overall (*N* = 280) included patients with myeloproliferative (*N* = 66), lymphocytic (*N* = 42), idiopathic (*N* = 155), other (*N* = 2) and unknown (*N* = 15) disease subtypes.

^b^ Maximum daily dose for maintenance therapy values over 60 mg were removed from the summary statistics, as these values likely reflected dosing for burst treatment episodes instead of maintenance therapy.

^c^ Other immunosuppressants or cytotoxic agents included chlorambucil, cyclosporine, dexpramipexole, etoposide, interferon-alpha, pegylated-interferon, ruxolitinib, tofacitinib, vincristine, leflunomide and mycophenolate.

^d^ Other biologics included omalizumab and reslizumab.

**Figure 2:**
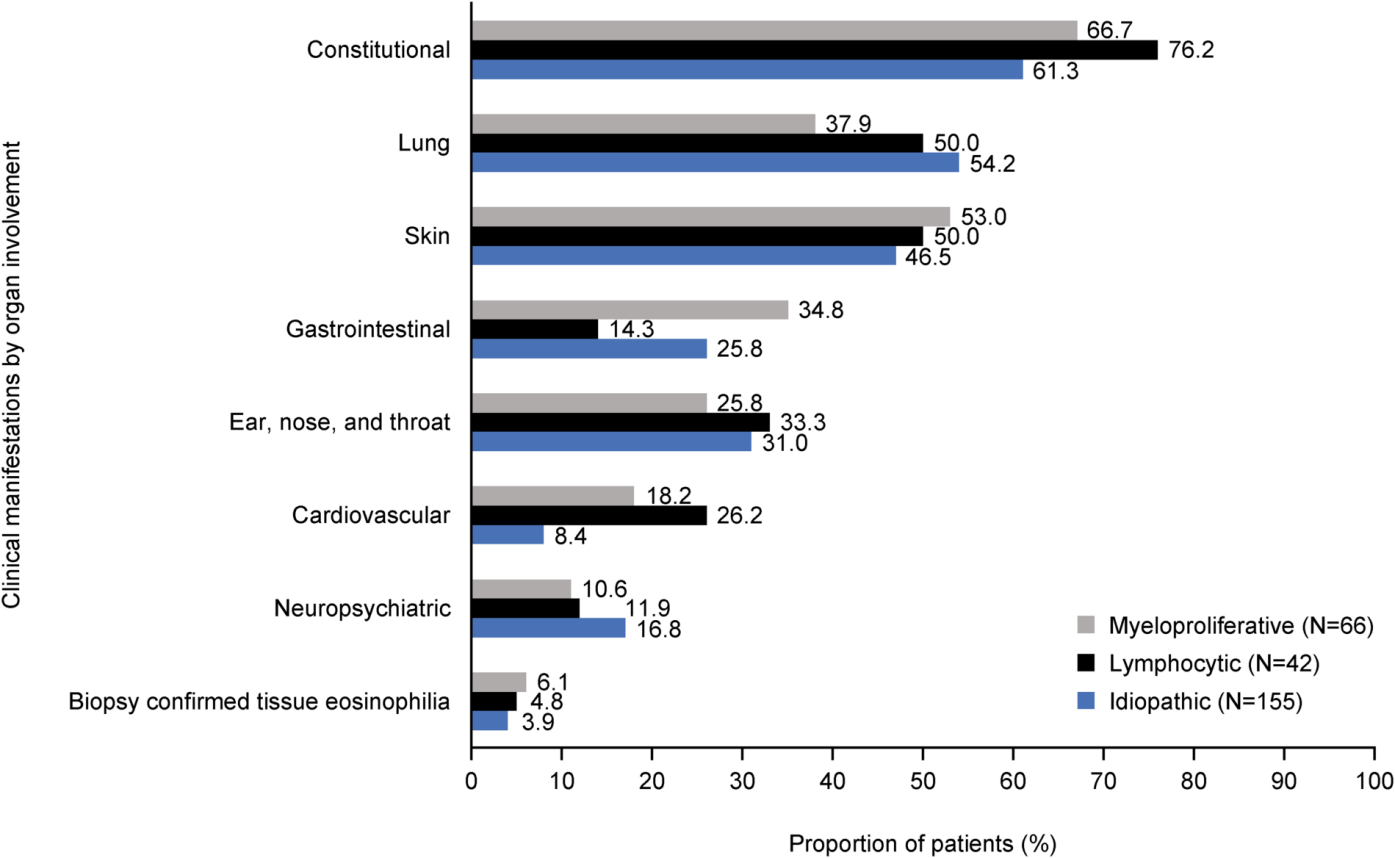
Clinical manifestations* by organ involvement across HES subtypes*.* *Clinical manifestations were assessed between index date and EOF (i.e., last physician encounter or death). Index date was defined as the date of a patient's earliest visit with their physician between January 2015 and December 2019 on or after the patient's HES diagnosis. Categories are ordered by the highest to lowest frequency of clinical manifestations in any HES subtype. EOF, end of follow-up; HES, hypereosinophilic syndrome.

A median (IQR) of 3.0 (1.0, 5.0) distinct clinical manifestations was observed for each HES subtype. The patterns of organ involvement varied across subtypes but constitutional, lung and skin manifestations were common across all (Figure 2). Gastrointestinal involvement was highest in M-HES (34.8%), cardiovascular involvement was highest in L-HES (26.2%) and neuropsychiatric involvement was highest in I-HES (16.8%). The most common distinct clinical manifestations were fatigue (54.2% I-HES, 52.4% L-HES and 42.4% M-HES), skin itch (36.4% M-HES, 35.7% L-HES and 33.5% I-HES) and pain (31.0% L-HES, 30.3% M-HES and 27.1% I-HES; Supplementary Table 2). These clinical manifestations were most commonly of moderate severity in each of the HES subtypes (moderate fatigue: 71.4% M-HES, 63.6% L-HES and 63.1% I-HES; moderate skin itch: 53.3% L-HES, 50.0% M-HES, 42.3% I-HES; and moderate pain: 80.0% M-HES, 73.8% I-HES, 69.2% L-HES; Supplementary Table 3).

The pattern of flare frequency and length, as well as clinical response, differed between subtypes (Table 4). The proportion of patients who experienced ≥1 flare/s was highest in the L-HES subtype (33.3%) and lowest in the M-HES subtype (18.2%). However, median flare duration was longest for M-HES (3.8 months) and shortest for I-HES (1.6 months). Clinical response rates were highest for I-HES (75.5%) than other subtypes (63.6%–66.7%) but with longer median time to first response with I-HES (13.9 months) than L-HES (7.2 months) or M-HES (8.2 months). Over 6 years of follow-up, mean FFS was 4.91 years overall, and 5.05, 4.24 and 5.04 years with the M-HES, L-HES and I-HES subtypes, respectively (Supplementary Figure 1). Over the same follow-up period, there were 6 deaths across all subtypes (Supplementary Figure 2).

**Table 4:** Clinical outcomes across HES subtypes.

Clinical outcomes	Overall (*N* = 280)	Myeloproliferative (*N* = 66)	Lymphocytic (*N* = 42)	Idiopathic (*N* = 155)
Flares (between index and EOF)^a^				
Patients who experienced a flare, *n* (%)	64 (22.9)	12 (18.2)	14 (33.3)	34 (21.9)
Number of flares per year,^b^ median (IQR)	0.4 (0.3, 0.7)	0.4 (0.3, 0.7)	0.4 (0.3, 0.6)	0.4 (0.3, 0.7)
Duration of flare(s), cumulative,^c^ median (IQR), months	2.1 (0.9, 3.9)	3.8 (1.0, 5.1)	2.2 (1.6, 4.0)	1.6 (0.9, 3.3)
Time to first flare from diagnosis, median (IQR), months	18.4 (10.1, 36.1)	16.8 (10.1, 28.0)	22.7 (9.7, 40.8)	23.0 (11.1, 36.5)
Treatment responses (between diagnosis and EOF)^d^				
Patients who experienced a response, *n* (%)	200 (71.4)	42 (63.6)	28 (66.7)	117 (75.5)
Duration of response(s), cumulative, ^e^ median (IQR), months	13.4 (4.8, 24.0)	15.4 (5.5, 25.7)	12.0 (2.9, 22.6)	12.2 (5.1, 25.8)
Time from diagnosis to first response,^f^ median (IQR), months	10.8 (4.1, 21.8)	8.2 (3.0, 19.5)	7.2 (3.1, 15.0)	13.9 (4.8, 24.0)
Patients who experienced a complete response, *n* (%)	113 (40.4)	22 (33.3)	19 (45.2)	68 (43.9)
Patients who experienced a partial response, *n* (%)	89 (31.8)	20 (30.3)	9 (21.4)	51 (32.9)

EOF, end of follow-up; HES, hypereosinophilic syndrome; IQR, interquartile range.

^a^ A flare was defined as the worsening of HES-related clinical symptoms or blood eosinophil counts requiring therapy escalation (e.g., increase in current therapy dosage or addition of new therapy).

^b^ Calculated from patients with ≥1 flare.

^c^ Calculated as the sum of durations for all reported flares.

^d^ Responses included both complete and partial responses. Complete response: physician-reported improved/resolved symptoms and blood eosinophil counts in the normal range (≤500 cells/µl). Partial response: physician-reported improved symptoms and blood eosinophil counts, where blood eosinophil count is not yet in the normal range (≤500 cells/µl) and the patient requires additional therapy. A small number of patients who had >1 different responses reported had both a complete response and a separate response reported.

^e^ Calculated as the sum of durations of response for all reported responses.

^f^ Among patients with ≥1 response.

HES-related hospitalizations were highest for L-HES (45.2%) than other subtypes (25.8%–30.3%) and the mean length of stay was longest with L-HES at 13.6 days, compared with 10.2 and 11.2 days for I-HES and M-HES, respectively (Supplementary Table 4). Emergency department visits were also most common with L-HES (47.6%) than other subtypes (22.6%–24.2%). The proportions reporting outpatient visits were similar (88%) across subtypes.

### 3.2 Characteristics and outcomes of patients with HES who received biologics

A total of 123 (43.9%) patients received biologics between HES diagnosis and end of follow-up. These patients had a mean age of 39.2 years at diagnosis, 58.5% were male and median blood eosinophil count was 1,000 cells/µl (Table 5). Over half of patients receiving biologics had I-HES (55.3%), while 22.0% had L-HES and 18.7% had M-HES. Asthma (56.9%), nasal polyps (48.8%), anxiety/depression (46.3%), hypertension (35.8%) and vasculitis (23.6%) were the most common comorbidities reported in patients receiving biologics (Table 6).

**Table 5:** Demographics and clinical characteristics of patients receiving biologics.

Patient baseline characteristics	Patients receiving biologics (*n* = 123)
Patient country, *n* (%)	
France	30 (24.4)
Germany	17 (13.8)
Italy	25 (20.3)
Spain	20 (16.3)
UK	31 (25.2)
Age at HES diagnosis, years, mean (SD)	39.2 (15.6)
Age on index date^a^, years, mean (SD)	41.4 (15.3)
6–11 years, *n* (%)	4 (3.3)
12–17 years, *n* (%)	5 (4.1)
Male, *n* (%)	72 (58.5)
Year of diagnosis, *n* (%)	
2014 or before	24 (19.5)
2015–2019	99 (80.5)
Disease duration^b^, years, median (IQR)	3.0 (1.8, 5.8)
Length of follow-up^c^, years, median (IQR)	2.5 (1.6, 3.8)
Blood eosinophil count, cells/µl, median (IQR)^d^	1,000.0 (450.0, 2,500.0)
Disease subtype, *n* (%)	
Myeloproliferative	23 (18.7)
Lymphoproliferative	27 (22.0)
Idiopathic	68 (55.3)
Unknown	5 (4.1)

^a^ Index date was defined as the date of a patient's earliest visit with their physician between January 2015 and December 2019 on or after the patient's HES diagnosis.

^b^ Disease duration was calculated as the time between HES diagnosis and EOF (i.e., last physician encounter or death).

^c^ Length of follow-up was calculated as the time between index date and EOF (i.e., last physician encounter or death).

^d^ Data available for *n* = 106 (86.2%) patients who received biologics. Physicians reported the most recent documented lab test value between the diagnosis date and index date.

**Table 6:** Comorbidities of patients receiving biologics.

Comorbidities,^a^ *n* (%)	Patients receiving biologics (*n* = 123)
Asthma	70 (56.9)
Nasal polyps	60 (48.8)
Anxiety or depression	57 (46.3)
Hypertension	44 (35.8)
Vasculitis	29 (23.6)
Lower respiratory disease(s)^b^	23 (18.7)
Obesity	18 (14.6)
Diabetes	17 (13.8)
Glomerulonephritis	15 (12.2)
Osteoporosis	14 (11.4)
Liver disease	13 (10.6)
Any cancer	11 (8.9)
Rheumatoid arthritis	6 (4.9)
Cerebrovascular disease	2 (1.6)
Leukemia	2 (1.6)
Other^c^	3 (2.4)

COPD, chronic obstructive pulmonary disease; EOF, end of follow-up; HES, hypereosinophilic syndrome; IQR, interquartile range; SD, standard deviation.

^a^ Comorbidities, and cancer diagnoses were assessed between HES diagnosis and EOF (i.e., last physician encounter or death).

^b^ Other than asthma and COPD.

^c^ Other reported comorbidities included allergy (*n* = 1), angioedema (*n* = 1), colitis (*n* = 1), esophagitis (*n* = 1), eosinophilic gastroenteritis (*n* = 1), restriction (*n* = 1) and rhinitis (*n* = 2).

In total, 41.5% of patients with HES receiving biologics were hospitalized between the index date and end of follow-up, with an average stay of >11 days (Supplementary Table 5). Thirty-nine percent of patients required an emergency department visit and 89.4% attended outpatient visits between the index date and end of follow-up.

Clinical manifestations, OCS use and clinical outcomes were analyzed in the pre-biologic and post-biologic periods among patients who received biologics and who had non-missing dates for ≥1 biologics record (*n* = 107). The incidence of fatigue was lower in the 12-month post-biologic period (8.4%) than the pre-biologic period (15.9%), whereas the incidence of gastrointestinal manifestations, itching and pain were slightly higher in the post-biologic period (11.2%, 10.3% and 8.4%, respectively) than in the pre-biologic period (7.5%, 6.5% and 2.8%, respectively; Table 7). It should be noted, however, that patient numbers for each of these clinical manifestations were low.

**Table 7:** Clinical manifestations, OCS reduction and clinical outcomes 12-months pre-/post-biologics.

Clinical manifestations and outcomes	Patients with HES who received biologics (*n* = 107)^a^	
	≤12 months pre-biologics^b^^,^^c^	≤12 months post-biologics^b^^,^^c^
Patients with manifestations, *n* (%) [95% CI]		
Fatigue	17 (15.9) [9.0, 22.8]	9 (8.4) [3.2, 13.7]
Asthma	11 (10.3) [4.5, 16.0]	12 (11.2) [5.2, 17.2]
Gastrointestinal^d^	8 (7.5) [2.5, 12.5]	12 (11.2) [5.2, 17.2]
Itch	7 (6.5) [1.9, 11.2]	11 (10.3) [4.5, 16.0]
Shortness of breath	5 (4.7) [0.7, 8.7]	5 (4.7) [0.7, 8.7]
Cardiovascular^e^	4 (3.7) [0.1, 7.3]	6 (5.6) [1.2, 10.0]
Pain	3 (2.8) [0, 5.9]	9 (8.4) [3.2, 13.7]
Rash	2 (1.9) [0, 4.4]	4 (3.7) [0.1, 7.3]
OCS use		
Annualized rate of OCS prescription,^f^ PPY	0.44	0.19
Patients with ≥1 OCS prescriptions,^f^ *n* (%) [95% CI]	34 (31.8) [23.0, 40.6]	12 (11.2) [5.2, 17.2]
Clinical outcomes, *n* (%) [95% CI]		
Patients with ≥1 response,^g^	7 (6.5) [1.9, 11.2]	25 (23.4) [15.3, 31.4]
Patients with ≥1 flare,^h^	13 (12.1) [6.0, 18.3]	12 (11.2) [5.2, 17.2]

CI, confidence interval; EOF, end of follow-up; HES, hypereosinophilic syndrome; OCS, oral corticosteroid; PPY, per person-year.

^a^ 123 out of 280 patients received biologics between HES diagnosis and EOF. 107 out of those 123 patients had non-missing dates for ≥1 biologics record. This analysis is out of those 107 patients.

^b^ The pre-biologics period was defined as 12 months before or on the initiation of biologics. The post-biologics period was defined as 12 months after the initiation of biologics.

^c^ For the pre-biologics period, only OCS prescriptions and person-years after HES diagnosis were included. For the post-biologics period, patients were censored if they died or had <12 months of follow-up period after biologics initiation.

^d^ Includes diarrhea, abdominal pain, nausea/vomiting, difficulty in swallowing food.

^e^ Includes cardiomyopathy, heart failure, thromboembolism, ischemic heart disease, valvular disease and arterial hypertension.

^f^ OCS prescriptions between HES diagnosis and EOF (i.e., last physician or death) were assessed.

^g^ Responses included both complete and partial responses. A complete response was defined as having physician-reported improved or resolved symptoms and normal blood eosinophil counts (≤0.5 × 10^9 ^/L). A partial response was defined as having physician-reported improved symptoms and blood eosinophil counts, where blood eosinophil counts is not yet in the normal range and the patient still requires additional therapy. Responses were assessed between HES diagnosis date and EOF (i.e., last physician encounter or death). A small share of patients who had >1 different responses reported had both a complete response and a separate response reported.

^h^ A flare was defined as the worsening of HES-related clinical symptoms or blood eosinophil counts requiring therapy escalation (e.g., increase in the dose of the current therapy or addition of new therapy). Flares were assessed between index date and EOF (i.e., last physician encounter or death). Index date was defined as the date of a patient's earliest visit with their physician between January 2015 and December 2019 on or after the patient's HES diagnosis.

The annualized rate of OCS prescriptions was reduced by 56.8% from 0.44 to 0.19 per person-year and the proportion of patients with ≥1 OCS prescription decreased by 64.7% (31.8% pre-biologics and 11.2% post biologics; Table 7). In terms of clinical outcomes, the proportion of patients with ≥1 response (complete or partial) was 3.6 times higher in the post-biologics period (23.4%) than in the pre-biologics period (6.5%), while the proportion of patients experiencing ≥1 flare was similar in the pre- and post-biologics periods (12.1% and 11.2%, respectively; Table 7).

## 4 Discussion

This subgroup analysis of real-world European data provides new insights into patient demographics, HES disease characteristics, treatment patterns and outcomes across different HES subtypes, raising awareness and adding to existing limited data. The analysis also provided real-world data regarding biologic use and treatment outcomes, for which there are limited data. This analysis indicated that patients with I-HES were more treatment responsive compared with patients with L-HES and M-HES, with a greater proportion of patients achieving a clinical response. This is consistent with a previous retrospective chart review study, which indicated that M-HES and L-HES had significantly worse odds of achieving a clinical response with OCS compared with I-HES (odds ratio [95% confidence interval] 0.34 [0.15–0.81], *p* = 0.015 for L-HES and 0.013 [0.0013–0.118, *p* = 0.0001 for M-HES) (13). There are nuances in the data from the current analysis, as the cumulative duration of responses was highest for M-HES, the proportion with complete responses was similar for I-HES and L-HES but lower for M-HES, and time to first response was longer for I-HES than the other subtypes. However, despite these factors, results indicate I-HES may be more treatment responsive than the other subtypes. Additionally, a lower proportion of patients in the current study with I-HES needed hospital or emergency department visits than patients with L-HES and M-HES, suggesting I-HES may be less severe than the other subtypes.

All HES subtypes have a substantial burden of disease, with constitutional; lung; skin; gastrointestinal; ear, nose and throat (ENT); cardiovascular; and neuropsychiatric issues affecting 8.4%–76.2% of patients in this study. This is consistent with a previous retrospective study of 188 patients with HES, which identified skin, pulmonary and gastrointestinal adverse impacts as being the most common clinical manifestations (28). The current analysis identified cardiovascular manifestations in 8% of patients with I-HES and up to 26% in those with L-HES; this is consistent with a case study review, which reported cardiac, rather than cardiovascular, manifestations in 12.9% of patients with L-HES, 14.9% with M-HES and 22.4% with I-HES (9).

There were also differences between HES subtypes in the prevalence of clinical manifestations within the organ domains of constitutional, cardiovascular, gastrointestinal and lung in this study. A higher proportion of patients with L-HES experienced constitutional manifestations such as pain, ENT manifestations such as sinus headache/facial pain/pressure and cardiovascular manifestations such as heart failure and thromboembolism, than with the other subtypes. Patients with M-HES experienced skin manifestations such as rash and gastrointestinal manifestations such as diarrhea, abdominal pain and nausea/vomiting slightly more frequently than patients with other subtypes. While overall, M-HES was the subtype with the highest prevalence of skin manifestations, the prevalence of hives/urticaria was highest in patients with L-HES. This corresponds with a case review study based on data from the French Reference Center for Hypereosinophilic Syndromes (CEREO), which reported eczema-like lesions, angioedema and urticaria in over 26% of patients with L-HES (24). However, while our data suggested that around half of patients with L-HES had skin manifestations, a retrospective study of 21 patients with L-HES initiated by the French Eosinophil Network, reported that 81% had skin manifestations (23). Finally, patients with I-HES had the highest prevalence of lung manifestations such as asthma and dyspnea and neuropsychiatric manifestations, such as sensory neuropathy, than the other subtypes.

Along with clinical manifestations, the high dosage of OCS received by patients in this study also underscores the substantial disease burden (mean maximum daily dose across all patients was 31.7 mg). The use of OCS, immunosuppressant/cytotoxic agents and biologics differed between HES subtypes. At baseline, the proportion receiving OCS was highest in patients with I-HES, the proportion receiving immunosuppressants/cytotoxic agents was highest in patients with M-HES and biologics were used by more patients with L-HES than the other subtypes. This is in accordance with findings from a previous review article that included individual case and aggregated data (9). This treatment pattern was seen with ongoing treatments at end of follow-up, with OCS being most used in I-HES, immunosuppressants/cytotoxic agents in M-HES and biologics in L-HES, with each prevalent drug class being used by approximately half of patients in their respective subtype.

Compared with the pre-biologic period, following post-biologic initiation there was a 56.8% reduction in the annualized rate of OCS prescriptions and a 64.7% decrease in the proportion of patients with ≥1 OCS prescription. Clinical response to treatment was also improved; 3.6 times as many patients had ≥1 clinical response in the ≤12-month post-biologic period, compared with pre-biologic. These findings are consistent with randomized controlled trials and open-label extensions that support the OCS-sparing effect, efficacy and safety of mepolizumab and benralizumab in the treatment of HES (18–20, 29, 30). The proportion of patients experiencing ≥1 flare was similar in the pre- and post-biologics periods, which may have reflected the fact that the study did not differentiate between different types of biologics used, including anti-IL-5, anti-IL-5 receptor, anti-cluster of differentiation (CD)20, anti-CD52, anti-IL-4 and anti-IL-13 therapies. A range of studies, however, have demonstrated that biologics targeting IL-5 or the IL-5 receptor can significantly reduce flares in HES (18, 25, 26, 31, 32). Additionally, there are clear knowledge gaps in terms of the efficacy of biologic therapies targeting eosinophilic inflammation in different HES subtypes (15). Therefore, examining the impact of different biologics on clinical outcomes in each HES subtypes, including those targeting IL-5/IL-5 receptor, would be an interesting topic to explore in future studies.

A strength of this study is the real-world nature of the data, which were collected across five European countries, meaning the findings may be generalizable across these healthcare systems and potentially more widely. Limitations of the study include that these were descriptive *post hoc* analyses with no statistical comparisons made, and there was no control population for the characterization of the biologics group. Therefore, the results should be seen as providing preliminary insights with the goal of providing hypotheses for further investigations.

## 5 Conclusion

Different HES subtypes are associated with differing disease burdens, treatment patterns and clinical outcomes. Biologic use appeared to be associated with numerically reduced OCS use and improved clinical responses, although these findings were not stratified by HES subtype. To improve patient care, further research is needed to optimize awareness regarding the differing clinical needs associated with each HES subtype and further characterize the impact of different biologic treatment across different subtypes.

## Data Availability

The original contributions presented in the study are included in the article/Supplementary Material, further inquiries can be directed to the corresponding author.
