# Supplementary material for: Steroid-sparing benefits of biologic use in hypereosinophilic syndrome and substantial disease burden across subtypes
Source: Front Allergy. 2025 May 23;6:1605397. doi: 10.3389/falgy.2025.1605397 (PMC12143263; doi:10.3389/falgy.2025.1605397)
Supplement: Supplementary file 1 [file Supplementaryfile1.docx]

**Supplementary Material**

**Supplementary Figure 1.** Real-world flare-free survival* across HES subtypes


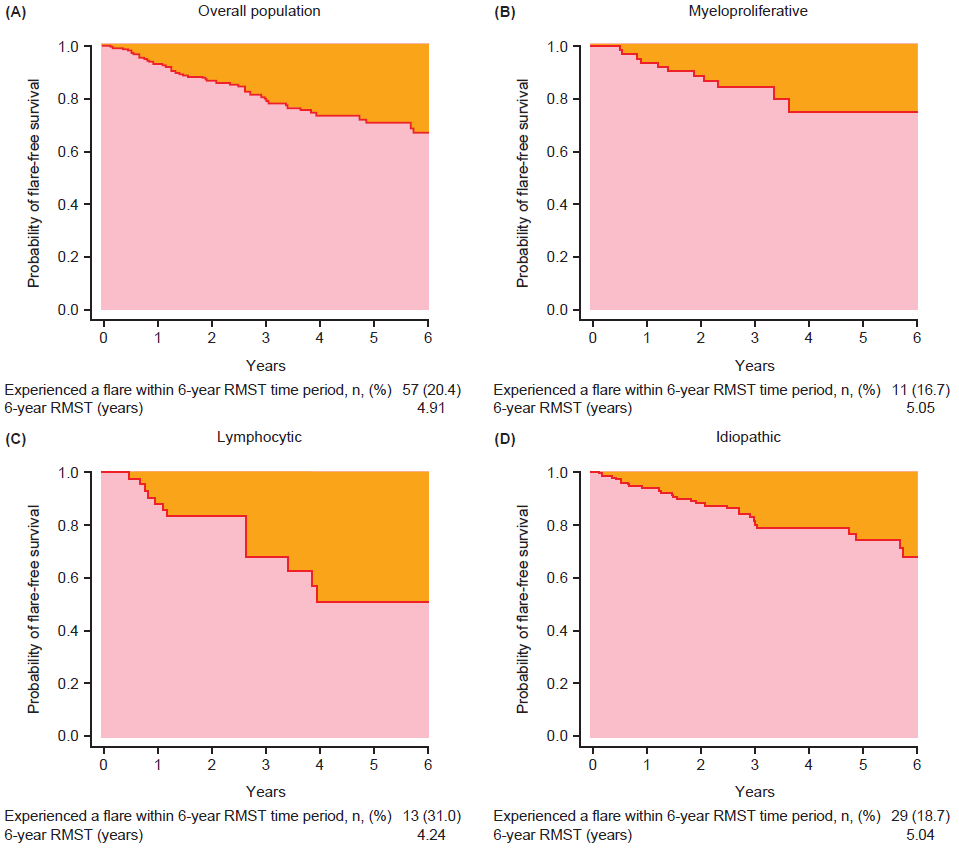


*Real-world flare-free survival was assessed over the 6 years after the diagnosis date using KM analysis. Patients were censored at EOF or at 6 years after the diagnosis date, whichever came first. Because the incidence of flares was observed to be <50%, the median survival time could not be estimated and RMST was reported instead. RMST is a measure of the average event-free survival time and is estimated by the area under the KM curve during a specified period of time.

EOF, end of follow-up; HES, hypereosinophilic syndrome; KM, Kaplan–Meier; RMST, restricted mean survival time.

**Supplementary Figure 2.** Real-world overall survival* across HES subtypes


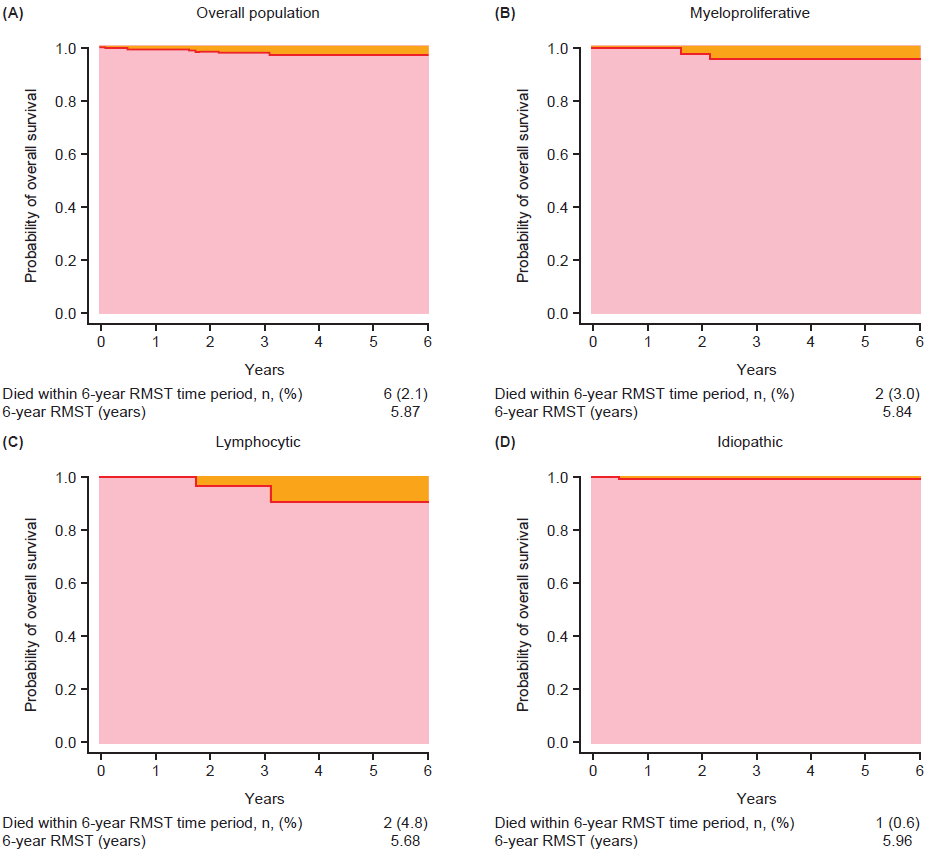


*Real-world overall survival was assessed over 6-year period after the diagnosis date by KM analysis. Patients were censored at EOF or at 6 years after the diagnosis date, whichever came first. Because the incidence of death was <50%, the median survival time could not be estimated and RMST, a measure of the average event-free survival time that is estimated by the area under the KM curve for a specified time period, was instead reported.

EOF, end of follow-up; HES, hypereosinophilic syndrome; KM, Kaplan–Meier; RMST, restricted mean survival time.

**Supplementary Table 1.** Ongoing* treatments at end of follow-up

|  | **Overall^†^ (N=280)** | **Myeloproliferative (N=66)** | **Lymphocytic (N=42)** | **Idiopathic (N=155)** |
| --- | --- | --- | --- | --- |
| **Oral corticosteroids, n (%)** | **125 (44.6)** | **27 (40.9)** | **15 (35.7)** | **80 (51.6)** |
| Prednisone or prednisolone | 97 (34.6) | 23 (34.8) | 9 (21.4) | 62 (40.0) |
| Methylprednisolone | 22 (7.9) | 2 (3.0) | 5 (11.9) | 15 (9.7) |
| Cortisone | 7 (2.5) | 2 (3.0) | 1 (2.4) | 4 (2.6) |
| **Most common immunosuppressants or  cytotoxic agents, n (%)** | **99 (35.4)** | **34 (51.5)** | **10 (23.8)** | **50 (32.3)** |
| Imatinib mesylate | 38 (13.6) | 19 (28.8) | 0 | 17 (11.0) |
| Azathioprine | 17 (6.1) | 1 (1.5) | 3 (7.1) | 13 (8.4) |
| Methotrexate | 12 (4.3) | 5 (7.6) | 1 (2.4) | 4 (2.6) |
| Hydroxyurea | 9 (3.2) | 3 (4.5) | 0 | 5 (3.2) |
| Cyclosporine | 6 (2.1) | 2 (3.0) | 1 (2.4) | 3 (1.9) |
| Other^‡^ | 20 (7.1) | 4 (6.1) | 5 (11.9) | 11 (7.1) |
| **Most common biologics, n (%)** | **97 (34.6)** | **15 (22.7)** | **20 (47.6)** | **58 (37.4)** |
| Mepolizumab | 32 (11.4) | 8 (12.1) | 7 (16.7) | 15 (9.7) |
| Benralizumab | 27 (9.6) | 4 (6.1) | 7 (16.7) | 15 (9.7) |
| Rituximab | 16 (5.7) | 3 (4.5) | 4 (9.5) | 8 (5.2) |
| Alemtuzumab | 15 (5.4) | 4 (6.1) | 1 (2.4) | 10 (6.5) |
| Dupilumab | 14 (5.0) | 2 (3.0) | 1 (2.4) | 11 (7.1) |
| Other^§^ | 14 (5.0) | 1 (1.5) | 6 (14.3) | 7 (4.5) |

*Ongoing treatments are treatments that the patient was indicated as taking at EOF; ^†^overall (N=280) included patients with myeloproliferative (N=66), lymphocytic (N=42), idiopathic (N=155), other (N=2) and unknown (N=15) disease subtypes; ^‡^other immunosuppressants included chlorambucil, cyclophosphamide, etoposide, interferon-alpha, pegylated-interferon, ruxolitinib, tofacitinib, and leflunomide; ^§^other biologics included omalizumab and reslizumab.
EOF, end of follow-up.

**Supplementary Table 2**. Specific clinical manifestations affecting more than 5% of patients in a single HES subtype

| **Clinical manifestations by organ involvement^†^, n (%)** | **Overall^*^ (N=280)** | **Myeloproliferative (N=66)** | **Lymphocytic (N=42)** | **Idiopathic (N=155)** |
| --- | --- | --- | --- | --- |
| **Constitutional** |  |  |  |  |
| Fatigue | 139 (49.6) | 28 (42.4) | 22 (52.4) | 84 (54.2) |
| Pain | 79 (28.2) | 20 (30.3) | 13 (31.0) | 42 (27.1) |
| Chills/sweats | 37 (13.2) | 10 (15.2) | 4 (9.5) | 21 (13.5) |
| Angioedema | 26 (9.3) | 10 (15.2) | 5 (11.9) | 10 (6.5) |
| **Lung** |  |  |  |  |
| Asthma | 66 (23.6) | 7 (10.6) | 9 (21.4) | 45 (29.0) |
| Dyspnea (shortness of breath) | 52 (18.6) | 10 (15.2) | 7 (16.7) | 31 (20.0) |
| Coughing | 42 (15.0) | 3 (4.5) | 8 (19.0) | 29 (18.7) |
| Pulmonary infiltration | 35 (12.5) | 4 (6.1) | 7 (16.7) | 23 (14.8) |
| Wheezing | 30 (10.7) | 7 (10.6) | 4 (9.5) | 17 (11.0) |
| **Skin** |  |  |  |  |
| Itch | 95 (33.9) | 24 (36.4) | 15 (35.7) | 52 (33.5) |
| Rash | 64 (22.9) | 20 (30.3) | 5 (11.9) | 36 (23.2) |
| Hives/urticaria | 32 (11.4) | 5 (7.6) | 8 (19.0) | 18 (11.6) |
| **ENT** |  |  |  |  |
| Nasal congestion | 46 (16.4) | 6 (9.1) | 4 (9.5) | 33 (21.3) |
| Sinus headache/facial pain/pressure | 30 (10.7) | 8 (12.1) | 9 (21.4) | 12 (7.7) |
| Postnasal drip | 28 (10.0) | 4 (6.1) | 4 (9.5) | 20 (12.9) |
| Purulent rhinorrhea | 11 (3.9) | 3 (4.5) | 1 (2.4) | 7 (4.5) |
| Ear fullness | 11 (3.9) | 1 (1.5) | 3 (7.1) | 7 (4.5) |
| **Gastrointestinal** |  |  |  |  |
| Diarrhea | 36 (12.9) | 10 (15.2) | 4 (9.5) | 20 (12.9) |
| Abdominal pain | 32 (11.4) | 9 (13.6) | 2 (4.8) | 20 (12.9) |
| Nausea/vomiting | 28 (10.0) | 9 (13.6) | 5 (11.9) | 13 (8.4) |
| Difficulty in swallowing food | 12 (4.3) | 2 (3.0) | 3 (7.1) | 6 (3.9) |
| **Neuropsychiatric** |  |  |  |  |
| Sensory neuropathy | 29 (10.4) | 6 (9.1) | 3 (7.1) | 20 (12.9) |
| **Cardiovascular** |  |  |  |  |
| Cardiomyopathy | 20 (7.1) | 7 (10.6) | 5 (11.9) | 8 (5.2) |
| Heart failure | 14 (5.0) | 3 (4.5) | 5 (11.9) | 5 (3.2) |
| Thromboembolism | 10 (3.6) | 2 (3.0) | 4 (9.5) | 4 (2.6) |

^*^Overall (N=280) included patients with myeloproliferative (N=66), lymphocytic (N=42), idiopathic (N=155), other (N=2) and unknown (N=15) disease subtypes; **^†^**clinical manifestations were assessed between index date and EOF (i.e. last physician encounter or death). Index date was defined as the date of a patient’s earliest visit with their physician between January 2015 and December 2019 on or after the patient’s HES diagnosis.
ENT, ear nose and throat; EOF, end of follow-up; HES, hypereosinophilic syndrome.

**Supplementary Table 3.** Severity at index date and change in severity of the three most common clinical manifestations of HES

| **Clinical manifestations by organ involvement,^2^ n (%)** | **Overall^1^ (N=280)** | **Myeloproliferative (N=66)** | **Lymphocytic (N=42)** | **Idiopathic (N=155)** |
| --- | --- | --- | --- | --- |
| **Fatigue** |  |  |  |  |
| Severity of manifestation at first documented occurrence on or after index date* |  |  |  |  |
| Mild | 28 (20.1) | 4 (14.3) | 4 (18.2) | 18 (21.4) |
| Moderate | 90 (64.7) | 20 (71.4) | 14 (63.6) | 53 (63.1) |
| Severe | 18 (12.9) | 4 (14.3) | 4 (18.2) | 10 (11.9) |
| Unknown | 3 (2.2) | 0 (0.0) | 0 (0.0) | 3 (3.6) |
| Overall change in severity^†^ |  |  |  |  |
| Worsening | 4 (14.3) | 0 (0.0) | 0 (0.0) | 4 (25.0) |
| Improvement | 8 (28.6) | 4 (57.1) | 1 (25.0) | 3 (18.8) |
| No change | 15 (53.6) | 3 (42.9) | 2 (50.0) | 9 (56.3) |
| Unknown | 1 (3.6) | 0 (0.0) | 1 (25.0) | 0 (0.0) |
| **Pain** |  |  |  |  |
| Severity of manifestation at first documented occurrence on or after index date* |  |  |  |  |
| Mild | 15 (19.0) | 3 (15.0) | 2 (15.4) | 8 (19.0) |
| Moderate | 58 (73.4) | 16 (80.0) | 9 (69.2) | 31 (73.8) |
| Severe | 5 (6.3) | 1 (5.0) | 2 (15.4) | 2 (4.8) |
| Unknown | 1 (1.3) | 0 (0.0) | 0 (0.0) | 1 (2.4) |
| Overall change in severity^†^ |  |  |  |  |
| Worsening | 0 (0.0) | 0 (0.0) | 0 (0.0) | 0 (0.0) |
| Improvement | 3 (15.0) | 2 (22.2) | 0 (0.0) | 1 (14.3) |
| No change | 17 (85.0) | 7 (77.8) | 3 (100.0) | 6 (85.7) |
| Unknown | 0 (0.0) | 0 (0.0) | 0 (0.0) | 0 (0.0) |
| **Skin itch** |  |  |  |  |
| Severity of manifestation at first documented occurrence on or after index date* |  |  |  |  |
| Mild | 29 (30.5) | 7 (29.2) | 3 (20.0) | 19 (36.5) |
| Moderate | 44 (46.3) | 12 (50.0) | 8 (53.3) | 22 (42.3) |
| Severe | 18 (18.9) | 5 (20.8) | 3 (20.0) | 9 (17.3) |
| Unknown | 4 (4.2) | 0 (0.0) | 1 (6.7) | 2 (3.8) |
| Overall change in severity^†^ |  |  |  |  |
| Worsening | 1 (5.6) | 0 (0.0) | 0 (0.0) | 1 (11.1) |
| Improvement | 6 (33.3) | 4 (57.1) | 2 (100.0) | 0 (0.0) |
| No change | 10 (55.6) | 3 (42.9) | 0 (0.0) | 7 (77.8) |
| Unknown | 1 (5.6) | 0 (0.0) | 0 (0.0) | 1 (11.1) |

*Severity was assessed using the following scale unless otherwise specified: Mild: present but minimal impact; Moderate: significant impact on daily activities; Severe: incapacitating; ^†^summary statistics for the overall change in severity of a given clinical manifestation were reported among the patients who had ≥2 occurrences of the clinical manifestation between index date and EOF; overall change in severity was defined as the change from the first reported occurrence to the last reported occurrence.
EOF, end of follow-up; HES, hypereosinophilic syndrome.

**Supplementary Table 4**. HES-related HCRU by HES subtype

|  | **Overall* (N=280)** | **Myeloproliferative (N=66)** | **Lymphocytic (N=42)** | **Idiopathic (N=155)** |
| --- | --- | --- | --- | --- |
| **Proportion of patients with any HES-related visits**^†^**, n (%)** |  |  |  |  |
| Hospitalizations | 85 (30.4) | 20 (30.3) | 19 (45.2) | 40 (25.8) |
| Average length of stay per hospitalization (days), mean (SD) [median] | 11.0 (9.4) [9.0] | 11.2 (10.2) [7.5] | 13.6 (14.5) [10.0] | 10.2 (5.5) [10.0] |
| Emergency department visits | 72 (25.7) | 16 (24.2) | 20 (47.6) | 35 (22.6) |
| Outpatient visits | 243 (86.8) | 58 (87.9) | 37 (88.1) | 137 (88.4) |
| **Mean number (SD) of HES-related visits (per person per year)**^†^ |  |  |  |  |
| Hospitalizations | 0.4 (1.2) | 0.4 (0.8) | 0.7 (1.1) | 0.3 (0.9) |
| Emergency departments visits | 0.3 (0.8) | 0.3 (0.7) | 0.5 (0.8) | 0.3 (0.8) |
| Overall outpatient visits | 4.3 (4.9) | 5.2 (5.4) | 4.3 (3.8) | 3.7 (3.1) |
| Unscheduled outpatient visits | 1.0 (3.3) | 0.9 (1.2) | 1.1 (1.2) | 0.8 (1.0) |
| **Proportion of patients with any occurrence of tests related to the complications and monitoring of adverse effects of using immunosuppressive medications**^†^**, n (%)** |  |  |  |  |
| Bone mineral density testing | 115 (41.1) | 33 (50.0) | 17 (40.5) | 63 (40.6) |
| Cataract removal | 17 (6.1) | 8 (12.1) | 2 (4.8) | 7 (4.5) |
| Imaging tests |  |  |  |  |
| CT scans | 95 (33.9) | 22 (33.3) | 17 (40.5) | 50 (32.3) |
| Chest radiographs | 64 (22.9) | 20 (30.3) | 10 (23.8) | 33 (21.3) |
| Echocardiograms or other cardiograms | 92 (32.9) | 27 (40.9) | 12 (28.6) | 50 (32.3) |
| Cardiac MRIs | 40 (14.3) | 10 (15.2) | 10 (23.8) | 20 (12.9) |
| Other imaging tests | 15 (5.4) | 2 (3.0) | 1 (2.4) | 10 (6.5) |
| **Mean number (SD) of tests related to the complications and monitoring of adverse effects of using immunosuppressive medications (per person per year)**^†^ |  |  |  |  |
| Bone mineral density testing | 0.3 (0.5) | 0.4 (0.6) | 0.3 (0.4) | 0.3 (0.5) |
| Cataract removal | 0.0 (0.2) | 0.1 (0.2) | 0.0 (0.2) | 0.0 (0.1) |
| Imaging tests |  |  |  |  |
| CT scans | 0.4 (0.9) | 0.4 (0.8) | 0.5 (0.8) | 0.3 (1.0) |
| Chest radiographs | 0.4 (1.7) | 0.5 (1.1) | 0.4 (1.3) | 0.4 (2.0) |
| Echocardiograms or other cardiograms | 0.4 (1.1) | 0.5 (0.7) | 0.3 (0.7) | 0.4 (1.3) |
| Cardiac MRIs | 0.1 (0.3) | 0.1 (0.2) | 0.2 (0.4) | 0.1 (0.4) |
| Other imaging tests | 0.1 (0.4) | 0.0 (0.1) | 0.0 (0.2) | 0.1 (0.6) |

^*^Overall (N=280) included patients with myeloproliferative (N=66), lymphocytic (N=42), idiopathic (N=155), other (N=2) and unknown (N=15) disease subtypes;
^†^HES-related visits and tests were assessed between index date and EOF (i.e. last physician encounter or death). Index date was defined as the date of a patient’s earliest visit with their physician between January 2015 and December 2019 on or after the patient’s HES diagnosis. The number of hospitalizations, emergency department visits, outpatient visits.

CT, computed tomography; EOF, end of follow-up; HCRU, healthcare resource utilization; HES, hypereosinophilic syndrome; MRI, magnetic resonance imaging; SD: standard deviation.

**Supplementary Table 5.** HES-related HCRU by biologic use^*^

| **HCRU** | **Patients receiving biologics  (n=123)** |
| --- | --- |
| **Proportion of patients with any HES-related visits^†^, n (%)** |  |
| Hospitalizations | 51 (41.5) |
| Average length of stay per hospitalization (days), mean (SD) [Median] | 11.3 (8.8) [10.0] |
| Emergency department visits | 48 (39.0) |
| Outpatient visits | 110 (89.4) |
| **Number of HES-related visits (per person per year), mean (SD) [median] ^†^** |  |
| Hospitalizations | 0.5 (0.8) [0.0] |
| Emergency department visits | 0.4 (0.7) [0.0] |
| Overall outpatient visits | 4.2 (4.3) [3.0] |
| Unscheduled outpatient visits | 1.0 (1.2),[0.7] |
| **Proportion of patients with any occurrence of tests related to the complications and monitoring of adverse effects of using immunosuppressive medications^†^, n (%)** |  |
| Bone mineral density testing | 53 (43.1) |
| Cataract removal | 10 (8.1) |
| Imaging tests |  |
| CT scans | 41 (33.3) |
| Chest radiographs | 29 (23.6) |
| Echocardiograms or other cardiograms | 40 (32.5) |
| Cardiac MRIs | 19 (15.4) |
| Other imaging tests | 4 (3.3) |
| **Number of tests related to the complications and monitoring  of adverse effects of using immunosuppressive medications  (per person per year), mean (SD) [median]^†^** |  |
| Bone mineral density testing | 0.3 (0.5) [0.0] |
| Cataract removal | 0.0 (0.2) [0.0] |
| Imaging tests |  |
| CT scans | 0.3 (0.6) [0.0] |
| Chest radiographs | 0.3 (0.9) [0.0] |
| Echocardiograms or other cardiograms | 0.3 (0.5) [0.0] |
| Cardiac MRIs | 0.1 (0.3) [0.0] |
| Other imaging tests | 0.0 (0.2) [0.0] |

^*^The overall population (N=280) included patients who were exposed to biologics (N=123) and not exposed to biologics (N=157) between HES diagnosis and EOF; **^†^**HES-related visits and tests were assessed between index date and EOF (i.e. last physician encounter or death). Index date was defined as the date of a patient’s earliest visit with their physician between January 2015 and December 2019 on or after the patient’s HES diagnosis. The number of hospitalizations, emergency department visits, outpatient visits, and tests were annualized.

CT, computer tomography; EOF, end of follow-up; HCRU, healthcare resource utilization; HES, hypereosinophilic syndrome; MRI, magnetic resonance imaging; SD, standard deviation.
